# Detection of a Novel Reassortant H9N9 Avian Influenza Virus in Free-Range Ducks in Bangladesh

**DOI:** 10.3390/v13122357

**Published:** 2021-11-24

**Authors:** Rabeh El-Shesheny, Jasmine C. M. Turner, David Walker, John Franks, Patrick Seiler, Subrata Barman, Mohammed M. Feeroz, Md Kamrul Hasan, Sharmin Akhtar, Nabanita Mukherjee, Lisa Kercher, Pamela McKenzie, Robert G. Webster, Richard J. Webby

**Affiliations:** 1 Department of Infectious Diseases, St. Jude Children’s Research Hospital, Memphis, TN 38105, USA; rabeh.elshesheny@stjude.org (R.E.-S.); jasmine.turner@stjude.org (J.C.M.T.); david.walker@stjude.org (D.W.); john.franks@stjude.org (J.F.); jon.seiler@stjude.org (P.S.); subrata.barman@stjude.org (S.B.); nabanita.mukherjee@stjude.org (N.M.); lisa.kercher@stjude.org (L.K.); pamela.mckenzie@stjude.org (P.M.); Robert.Webster@STJUDE.ORG (R.G.W.); 2 Center of Scientific Excellence for Influenza Viruses, National Research Centre, Giza 12622, Egypt; 3 Department of Zoology, Jahangirnagar University, Savar 1342, Bangladesh; feerozmm@yahoo.com (M.M.F.); mkhasan@ucdavis.edu (M.K.H.); sarumpazoolju@gmail.com (S.A.)

**Keywords:** avian influenza, free-range ducks, Bangladesh

## Abstract

Wild aquatic birds are the primary natural reservoir for influenza A viruses (IAVs). In this study, an A(H9N9) influenza A virus (A/duck/Bangladesh/44493/2020) was identified via routine surveillance in free-range domestic ducks in Bangladesh. Phylogenetic analysis of hemagglutinin showed that the H9N9 virus belonged to the Y439-like lineage. The HA gene had the highest nucleotide identity to A/Bean Goose (*Anser fabalis*)/South Korea/KNU 2019-16/2019 (H9N2). The other seven gene segments clustered within the Eurasian lineage.

## 1. Introduction

Influenza A viruses (IAVs) threaten the health of humans and animals worldwide. Avian influenza viruses (AIVs) are widespread in nature, with waterfowl serving as the primary reservoir from which transmission of influenza subtypes to domestic poultry occurs [1]. IAVs are classified based on surface glycoproteins hemagglutinin (HA) and neuraminidase (NA), with 18 HA subtypes (H1–H18) and 11 NA subtypes (N1–N11). To date, many combinations of HA (H1-16) and NA (N1-9) subtypes have been identified in wild birds.

Reassortment represents a major mechanism supporting the emergence and evolution of IAVs. Domestic ducks can perpetuate many AIV subtypes via asymptomatic shedding, which plays an important role in the epidemiology, dissemination, and reassortment of low-pathogenic avian influenza (LPAI) viruses [1]. Specifically, free-range ducks are considered to be a primary link for bidirectional transmission of AIVs between wild and domestic birds providing the interface that play role for AIV reassortment. Epidemiological data indicated that recent zoonotic H7N9 and H10N8 infections were caused by viruses that originated in domestic ducks and spread to chickens [2,3,4]. Our previous studies have also shown the role of free-range ducks in the spread of influenza viruses to other species in live-poultry markets in Bangladesh [5,6].

Highly pathogenic AIV (H5N1) was initially introduced into Bangladesh in 2007 and has since become endemic in poultry. Multiple clades of H5N1 Gs/Gd lineage viruses (Clades 2.2.2, 2.3.4.2, and 2.3.2.1a) were identified in Bangladeshi live-poultry markets (LPMs) [7,8]. Due to the continued circulation of H5N1 Gs/Gd lineage in LPMs along with LPAI viruses, reassortment between HPAI H5N1 and LPAI viruses was detected in live-poultry markets in Bangladesh [9]. A recent study detected H5Nx reassortants that had high virulence in mice and chickens but limited transmission between ferrets [10]. Adaptions and continuous reassortments can not only facilitate the spread of the IAVs but can also contribute to increased levels of genetic diversity.

H9N2 AIVs were widespread in poultry globally and sporadically detected in humans. As of October 2021, a total of 82 laboratory-confirmed human cases with H9N2 infections have been reported [11]. Based on the phylogenetic clustering of HA genes, H9N2 viruses can be divided to three main lineages: G1-like, Y280-like/BJ/94-like, and Y439-like. The G1-like lineage viruses circulate in Asia, the Middle East, and Africa. The Y280-like/BJ/94-like lineage viruses circulate in China. The Y439 lineage viruses circulate endemically in poultry in South Korea and have been isolated from wild birds and poultry in Europe, Asia, and South Africa [12]. H9N2 AIVs have been confirmed as donors of internal genes to AIVs with pandemic potential such as H5Nx, H7N9, and H10N8 AIVs, posing an enormous threat to both human and animal health.

Active surveillance for AIV in Bangladesh during the past decade led to the identification of several influenza virus subtypes, most abundantly H9N2. The first reported detection of H9N2 viruses of the G1 lineage in Bangladesh occurred in 2006 in a commercial breeder. Continued circulation of H9N2 viruses in Bangladesh have allowed for the accumulation of molecular markers that enhance viral replication in mammals [13]. Human infections with influenza H9N2 viruses were first detected in Bangladesh in 2011. The continued presence of these viruses at the human–poultry interface provides opportunities for H9N2 AIVs to donate significant genetic material to emerging zoonotic viruses, threatening both human health and the poultry industry. Here, we analyzed the phylogenetic and genetic properties of the newly identified reassortant H9N9 isolate from free-range domestic ducks in Tanguar Haor.

## 2. Materials and Methods

### 2.1. Sampling and Surveillance for AIVs

Active surveillance of poultry has been conducted in Bangladesh through collaborative efforts of the Center of Excellence for Influenza Research and Surveillance at St. Jude (Memphis, TN, USA) and Jahangirnagar University (Dhaka, Bangladesh) since 2008 in LBMs and since 2015 in Tanguar Haor, a wetland area in northeastern Bangladesh where local domestic ducks are reared and where birds winter during the migratory season. Swab and fecal samples were collected from various species of wild birds and from free-range ducks from the Tanguar Haor area. Samples were collected as previously described [5,14,15]. Samples were stored at ~4 °C in the field, moved to liquid nitrogen within one week of collection, and then shipped to the biosafety level 3 facilities of St. Jude Children’s Research Hospital in Memphis, TN, USA, for further analysis.

### 2.2. Virus Isolation from Samples

Samples were screened for the presence of AIV by real-time RT-PCR targeting the M gene by using TaqMan probes, 4x Fast Virus RT-PCR kit (Life Technologies, MD, USA), in ABI 7500 Fast PCR. Primer and probe sequences are as follows: forward primer: 5′-GACCRATCCTGTCACCTCTGAC-3′, reverse primer: 5′- AGGGCATTYTGGACAAAKCGTCTA-3′, probe: 5′-6FAM-TGCAGTCCTCGCTCACTGGGCACG-TAMRA-3′. Samples were classified as positive with a Ct value ≤ 36. All positive samples were inoculated in 10-day-old embryonated chicken eggs for virus isolation. Isolated viruses were incubated at 38 °C for 72 h and stored at 4 °C overnight. Hemagglutination assays (HA) of the allantoic fluids from the inoculated eggs were performed to screen for IAV according to World Health Organization (WHO) and World Organization for Animal Health (OIE) protocols [16,17].

### 2.3. Subtype Detection and Sequencing

This step was performed as previously described [10]. Briefly, viral RNA was extracted from allantoic fluid before conventional two-step reverse transcription–PCR was performed by using a SuperScript IV first-strand synthesis kit (Invitrogen) and the Uni12 influenza primer. Then, Phusion high-fidelity DNA polymerase (New England Biolabs, Ipswich, MA, USA) and Uni12/13 primers were used for multiplex PCR of all eight gene segments, and PCR products were purified. The staff of the Hartwell Center at St. Jude Children’s Research Hospital prepared the DNA libraries, which were then pooled and sequenced via 150 bp paired end reads by using an Illumina MiSeq personal genome sequencer. The sequencing reads were analyzed by using CLC Genomics Workbench, version 20 (CLC Bio, Qiagen, Hilden, Germany).

### 2.4. Phylogenetic Analysis

As in our previous study, all reference sequences were obtained from the NCBI Influenza Virus Resource (https://www.ncbi.nlm.nih.gov/genbank/) and GISAID (http://www.gisaid.org), accessed on 15 April 2021, and BLAST homology analysis of nucleic acids was performed on the NCBI and/or GISAID website. DNA Lasergene 15 and BioEdit7.0 [18] were used for multiple sequence alignment and genomic signature analysis using the ClustalW algorithm [19]. MEGA 7 was used for the phylogenetic tree reconstruction by applying the neighbor-joining method with Kimura’s two-parameter distance model and 1000 bootstrap replicates [20].

## 3. Results and Discussion

Active surveillance of poultry has been conducted in Bangladesh and several influenza virus subtypes, including highly pathogenic H5Nx and H9N2 and other low pathogenic avian influenza viruses, have been isolated [5,6,9,10,15,21]. On 22 February 2020, AIV virus was isolated from the swab sample of a Khaki Campbell duck and confirmed via sequencing to be an H9N9 virus. We performed deep sequencing to determine the complete genome sequence of the newly identified H9N9 virus (GenBank accession numbers MW749817.1 to MW749823.1).

We studied the genetic relationship between the H9N9 virus, other viruses isolated from Bangladesh, and other viruses available in GenBank. The HA genes shared 97.62% nucleotide identity with A/Bean Goose (Anser fabalis)/South Korea/KNU 2019-16/2019 (H9N2) (Table 1), and the NA gene shared 98.47% nucleotide identity with A/Anas platyrhynchos/South Korea/JB31-96/2019 (H11N9). Other internal genes shared high nucleotide identities with viruses isolated from Tanguar Haor, Bangladesh, during the same season (Table 1).

**Figure 1:**
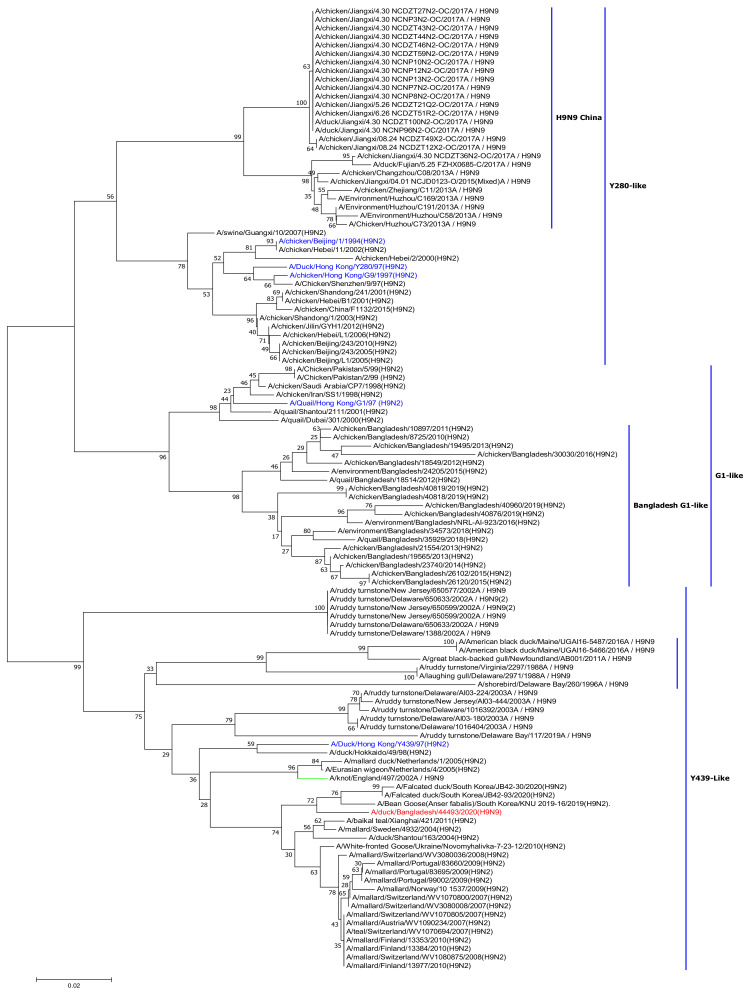
Phylogenetic tree of the HA gene. Phylogenetic analysis was performed using the neighbor-joining algorithm with the Kimura two-parameter model. The reliability of phylogenetic inference at each branch node was estimated by the bootstrap method with 1000 replications; evolutionary analyses were conducted in mega 7. The HA gene of H9N9 virus isolate in this study is marked in red and H9N2 reference strains are marked in blue.

To better understand the genetic relationship of the H9N9 virus to other influenza viruses, we conducted phylogenic analyses. The sequences of these viruses are available in GenBank and the GISAID database (https://www.gisaid.org, accessed on 15 April 2021). This analysis revealed that the HA sequence clustered with the Y439-like lineage and was most closely related to those of H9N2 viruses isolated from South Korea in 2019–2020 (Figure 1). The amino acid sequence of the HA of the H9N9 virus included a single arginine residue at the cleavage site of HA1 and HA2 (PAASDR↓GLF), indicating that it was a low pathogenic strain in the chicken host [22,23,24]. We examined the presence of N-linked glycosylation motifs by interrogating the proteins for N-(P-[S/T]-P) motifs, which indicate potential N-linked glycosylation sites. We identified seven potential glycosylation sites at positions 29–31 (NTS), 141–143 (NVT), 218–220 (NRT), 298–300 (NTT), 305–307 (NIS), 492–494 (NGT), and 551–553 (NGS).

We evaluated residues known to impact receptor-binding preference because mutations conveying a preference for human-type receptors could enable zoonotic transmission. Our isolate harbored HA markers (198 E, 226Q, and 228G), suggesting more efficient binding to avian receptors than to human receptors [25]. However, the H9N9 virus had the I155T mutation, which plays an important role in binding the H9N2 virus to the human-type receptor [26].

**Figure 2:**
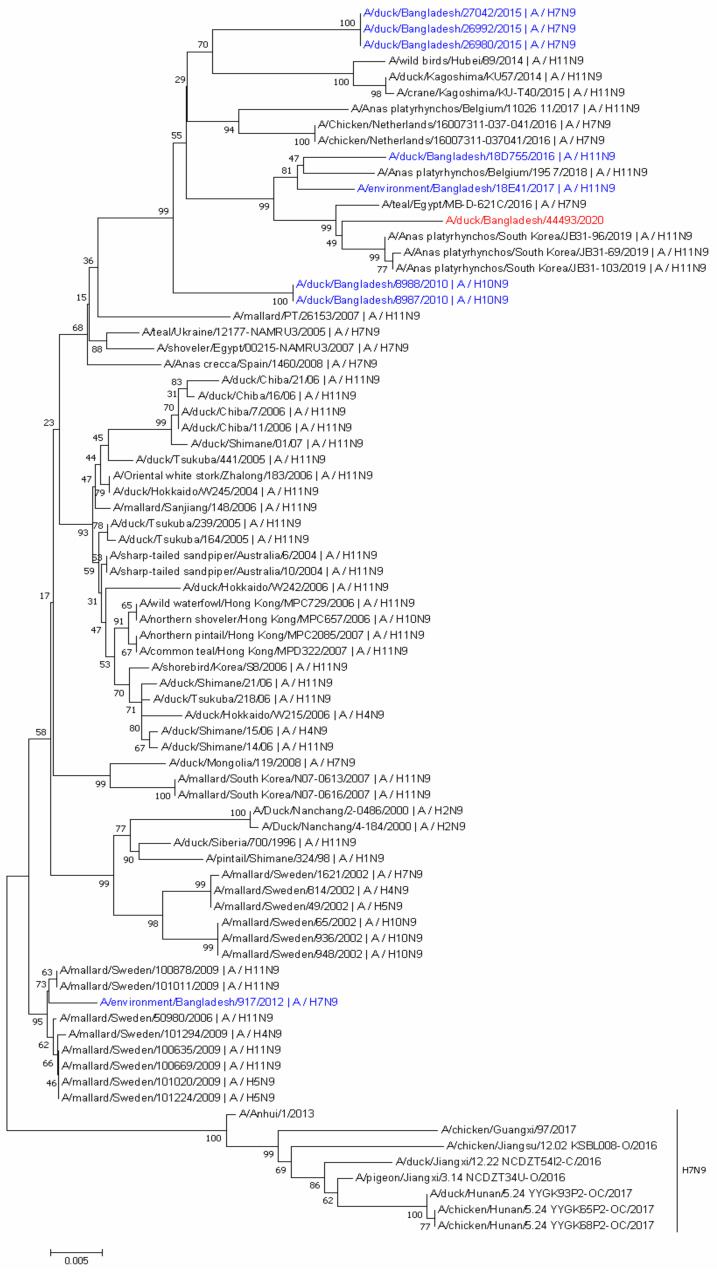
Phylogenetic tree of the NA gene. Phylogenetic analysis was performed using the neighbor-joining algorithm with the Kimura two-parameter model. The reliability of phylogenetic inference at each branch node was estimated by the bootstrap method with 1000 replications; evolutionary analyses were conducted in mega 7. The NA gene of H9N9 virus isolate in this study is marked in red. N9 gene of viruses isolated from Tanguar Haor area in Bangladesh are marked in blue.

Phylogenetic analysis of the H9N9 NA gene showed that it was most closely related to H11N9 AIVs isolated from South Korea in 2019 that belonged to the wider Eurasian lineage, also related to A/teal/Egypt/MB-D-621C/2016 (H7N9) (Figure 2). Additionally, the NA gene of H9N9 strain showed similarities with H11N9, H7N9, and H10N9 strains isolated from free-range ducks in Tanguar Haor in 2010, 2015, 2016, and 2017 [21]. This finding indicates that in Bangladesh, the NA genes may circulate between wild birds and free-range ducks. The N9 gene of the H9N9 isolate showed 93.5% nucleotide identity with A/Anhui/1/2013(H7N9), which caused human infections in China, and clustered within a different group (Figure 2). Our isolates did not display oseltamivir resistance markers E119, H275, R293, and N295 (N1 numbering).

**Figure 3:**
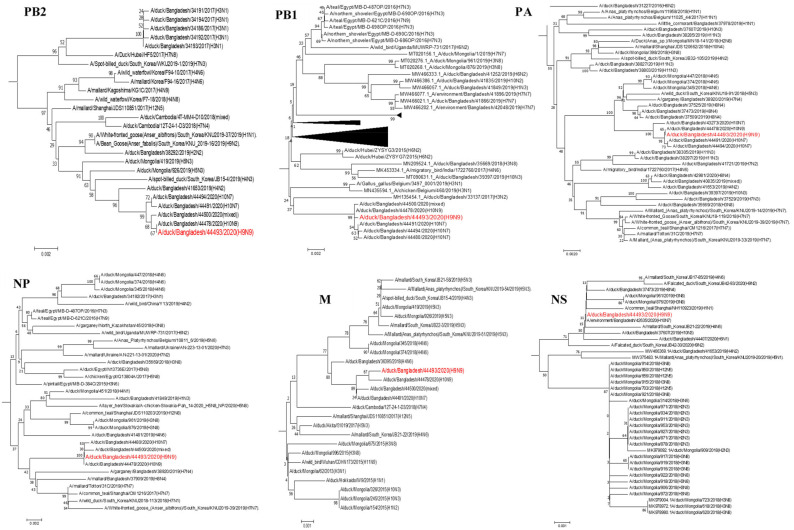
Phylogenetic trees of six internal genes of the isolated H9N9 virus. Phylogenetic trees were constructed for six internal gene segments using the limited homologous viruses. H9N9 virus isolate in this study is marked in red.

Phylogenetic analyses of the six internal genes (PB2, PB1, PA, NP, M, and NS) of the H9N9 isolate showed that it belonged to the Eurasian lineage (Figure 3). The E627K and D710N substitutions in the PB2 gene, which play an important role in the adaptation of AIVs to mammals [27], were not detected in our isolate. However, the PB2 amino acid substitutions L89V, G309D, and T339K, which increase replication in human lung epithelial cells and mice [28,29], were observed in the PB2 gene. The H9N9 isolate’s PA contained amino acid substitutions (Table 2), including the PA-A515T mutation, which may be associated with adaptation to mammals [30]. The PB1-F2 protein was variable in length, with PB1-F2 of H9N9 isolate being 90-aa long. It also had the N66S mutation, which increases virulence, replication efficiency, and the antiviral response in mice [31].

No substitutions associated with antiviral resistance to the adamantanes were identified. Our analyses showed that NS1 protein of H9N9 contains amino acid substitutions P42S and V149A (Table 2), and these mutations were found to be associated with virulence and pathogenicity in mammals.

**Figure 4:**
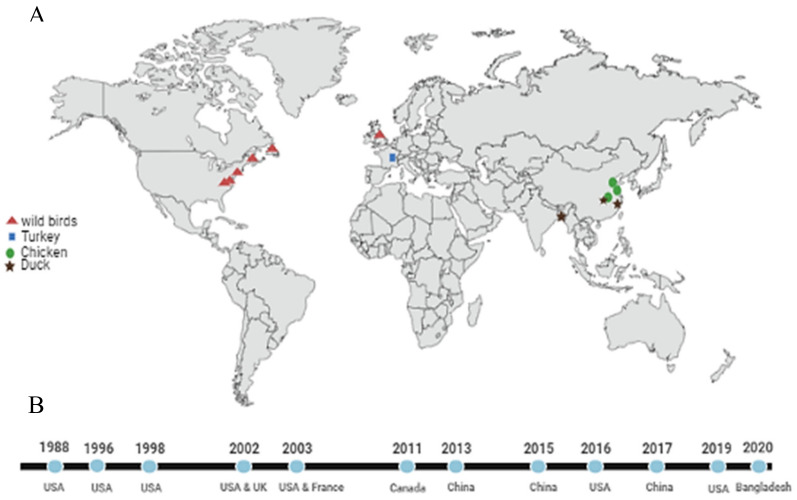
Emergence and distribution of H9N9 viruses from 1988 to 2020. (**A**) Distributions of H9N9 viruses in different countries and host range. (**B**) Detection timeline of H9N9 viruses.

H9N9 virus is a rarely isolated subtype among IAV, most being isolated from wild birds from North America (Figure 4). The first detections were in 1988 from ruddy turnstone and laughing gull from Virginia and Delaware Bay, respectively: A/ruddy turnstone/Virginia/2297/1988 (H9N9) and A/laughing gull/Delaware/2971/1988 (H9N9). Through longitudinal surveillance in Delaware Bay, we detected this subtype in ruddy turnstones from 1988–2019 (Figure 4B) [49]. This subtype was detected in England and France before 2013 (Figure 4). H9N9 was detected in China in 2013; the HA closely clustered with the H9N2 strains circulating in China at the time, but the NA genes clustered with the H7N9 viruses [50]. This pattern suggests that the H9N9 virus isolated from China may have originated from reassortment of H9N2 viruses with H7N9 viruses within a domestic avian host.

The phylogenetic trees of HA and NA indicate that the newly identified H9N9 is most genetically similar to H9N2 and H11N9 viruses isolated from South Korea rather than to Bangladeshi H9N2 viruses, suggesting that ancestors of this virus emerged among wild birds and were transmitted to Tanguar Haor’s free-range ducks. Wild aquatic birds are considered to be natural reservoirs for AIVs and are vital for AIV to evolve, be maintained, and spread. Bangladesh is in the Central Asian flyway and near the Eastern Asian-Australian and Black Sea-Mediterranean flyways. It also serves as a stopover site for migratory birds. We previously showed that interactions between wild birds and ducks play a role in reassortments and emerging new strains [5,6]. Two scenarios exist for the genesis of this virus, that it was generated elsewhere and arrived in Bangladesh with bird movement or that it arose in Bangladesh through local reassortment. The phylogenetic relationships of the genes of this virus with LPAI viruses isolated from Bangladesh led us to favor the latter possibility.

Controlling AIVs in Bangladesh requires the integration of multiple strategies including vaccination, active surveillance, biosecurity, and improved awareness of producers and traders. HPAIV vaccination strategies were introduced in Bangladesh in 2012 and vaccination against H9N2 has recently been approved [51].

When different viruses co-circulate in the same area, novel reassortants with variable phenotypes can emerge. In our surveillance, we detected other LPAI viruses from domestic duck and/or free-range ducks and wild birds in the Tanguar Haor area in Bangladesh, raising concern about the potential for the emergence of reassortant viruses. Evaluating the biologic properties of the newly identified H9N9 virus is crucial for increased knowledge of these viruses’ emergence and evolution. Such knowledge is vital to predict and fight potential pandemics that would threaten public health.

## Figures and Tables

**Table 1:** Comparison of nucleotide sequence identities of the eight influenza A virus (IAV) gene sequences for the virus isolated in this study (A/duck/Bangladesh/44493/2020 (H9N9)) and nearest virus homologs.

Gene *	Accession No.	Virus	% Identity
PB2	MT020147.1	A/duck/Mongolia/926/2019(H5N3)	99.1
	MW749040.1 ^†^	A/duck/Bangladesh/44478/2020(H10N9)	100
PB1	MN208036.1	A/northern shoveler/Egypt/MB-D-690C/2016(H7N3)	99.63
	MW749040.1	A/duck/Bangladesh/44478/2020(H10N9)	99.96
PA	MW188628.1	A/duck/Mongolia/447/2018(H4N6)	99.1
	MW749040.1	A/duck/Bangladesh/44478/2020(H10N9)	100
HA	EPI_ISL_418175	A/Bean Goose(Anser fabalis)/South Korea/KNU 2019-16/2019(H9N2)	97.62
NP	MN208011.1	A/teal/Egypt/MB-D-487OP/2016(H7N3)	98.72
	MW749040.1	A/duck/Bangladesh/44478/2020(H10N9)	100
NA	MW116667.1	A/Anas platyrhynchos/South Korea/JB31-96/2019(H11N9)	98.47
M	MW188600.1	A/duck/Mongolia/345/2018(H4N6)	99.21
	MW749040.1	A/duck/Bangladesh/44478/2020(H10N9)	99.80
NS	MT020282.1	A/duck/Mongolia/961/2019(H3N8)	99.77
	MW466161.1	A/environment/Bangladesh/42635/2020(H10N7)	100

* PB2, basic polymerase 2; PB1, basic polymerase 1; PA, acidic polymerase; HA, hemagglutinin; NP, nucleoprotein; NA, neuraminidase; MP, matrix protein; NS, nonstructural protein. ^†^ Nearest virus homologs to A/duck/Bangladesh/44493/2020 (H9N9) isolated from Tanguar Haor wetlands in Bangladesh.

**Table 2:** Assessment of molecular markers for zoonotic potential of the influenza A(H9N9) virus.

Viral Protein	Amino Acid	A/duck/Bangladesh/44493/2020	Functional Relevance	References
PB2	E627K	E	Mammalian host adaptation	[32]
	D701N	D	Increase polymerase activity and viral replication in mammalian cells	[33]
	L89V	V	Enhanced polymerase activity, increased virulence in mice	[28]
	G309D	D	Enhanced polymerase activity, increased virulence in mice	[28]
	T339K	K	Enhanced polymerase activity, increased virulence in mice	[28]
	A588V	A	Mammalian host adaptation	[34]
PB1-F2	N66S	S	Increases virulence, replication efficiency, and the antiviral response in mammals	[31,35]
PA	V100A	V	Contributed to the virulence and mammalian adaptation	
	S409N	S	Contributed to the virulence and mammalian adaptation	[36]
	A515T	T	Increased polymerase activity, increased virulence in mammals and birds	[30]
HA	E198D	E	Enhanced mammalian receptor binding	[37]
	Q234L	Q	Preferential binding to human Sialic acid α2–6 receptor	[38,39]
	G236S	G	Preferential binding to human Sialic acid α2–6 receptor	[38]
	I155T	T	Enhanced mammalian receptor binding	[40]
NA	E119V	E	Oseltamivir resistance	[41]
	H275Y	H	Oseltamivir resistance	[41]
	R293K	R	Oseltamivir resistance	[42]
	N295S	N	Oseltamivir resistance	[41]
M2	L26P	L	Reduced susceptibility to amantadine	[43,44]
	V27A/I	V	Reduced susceptibility to amantadine	[43,44]
	A30T	A	Reduced susceptibility to amantadine	[44,45]
	S31N	S	Reduced susceptibility to amantadine	[44,45]
	G34E	G	Reduced susceptibility to amantadine	[43]
NS1	P42S	S	Increased virulence and pathogenicity in mammals	[46]
	D92E	D	Increased virulence and pathogenicity in mammals	[47]
	V149A	A	Increased virulence and pathogenicity in mammals	[48]

## Data Availability

The raw data supporting the conclusions of this manuscript has been submitted to GenBank and will be made available by the authors, without undue reservation, to any qualified researcher.
